# Streamlining Ophthalmic Documentation With Anonymized, Fine-Tuned Language Models: Feasibility Study

**DOI:** 10.2196/72894

**Published:** 2025-11-26

**Authors:** Sebastian Arens, Quang Vinh Ngo, Anna Richling, Lucas Stürzbecher, Daniel Böhringer, Thomas Reinhard, Felix Heilmeyer

**Affiliations:** 1 Eye Center University Medical Center Freiburg Freiburg im Breisgau, Baden Wuerttemberg Germany; 2 Center for Digitization in Medicine University Medical Center Freiburg Freiburg im Breisgau, Baden Wuerttemberg Germany

**Keywords:** artificial intelligence, large language models, medical reports, epicrisis generation, documentation automation

## Abstract

**Background:**

The growing administrative burden on clinicians, particularly in medical documentation, contributes to burnout and may compromise patient safety. Recent advancements in generative artificial intelligence (AI) offer a promising solution to improve documentation processes and address these challenges.

**Objective:**

This study aims to evaluate the feasibility of using a fine-tuned OpenAI Curie model to automate the generation of medical report summaries (epicrises) in ophthalmology. By assessing the model’s performance through human and automated evaluations, this study seeks to determine its potential for reducing clinician workload while ensuring accuracy, usefulness, and compliance with regulatory requirements.

**Methods:**

A data set of around 60,000 anonymized medical letters was created using a custom algorithm to comply with General Data Protection Regulation guidelines. The Curie model was fine-tuned on this data set to generate epicrises from medical histories, diagnoses, and findings. The performance evaluation involved various human assessments and automated evaluations from 2 large language models (LLMs).

**Results:**

In the clinical context, 49.9% (384/769) of epicrises were evaluated as helpful or excellent, whereas only 25% (194/769) were considered disturbing. In a human (manual) evaluation, formal correctness was rated significantly higher than the neutral midpoint of 2.5 on the 4-point rating scale, as determined by a 1-sample Wilcoxon signed-rank test (mean 3.59, SD 0.85; *W*=1686; *P*<.001). Using paired *t* tests, we found a significant reduction in time, as correcting an AI epicrisis was faster than manually writing one (mean 109.52, SD 53.30 vs mean 54.25, SD 63.34 s; *t*_68_=3.39; *P*<.01). While medical accuracy and usefulness showed positive trends, these did not reach statistical significance when compared to the neutral midpoint (for medical accuracy, *W*= 7456; *P*=.08), for usefulness, *W*=7652.5; *P*=.18). Epicrises generated or corrected with AI were significantly shorter than manually written ones (mean 330.43, SD 115.42 vs mean 501.07, SD 243.50 characters; *t*_68_=–6.10; *P*<.001). Automated LLM assessments showed alignment with human ratings, with over 52% (356/679) and 66% (489/743) of responses in the top agreement categories, respectively. This supports overall consistency, though the comparison remains a proof of concept given methodological limitations.

**Conclusions:**

Our study demonstrates the technical and practical feasibility of introducing fine-tuned commercial LLMs into clinical practice. The AI-generated epicrises were formally and clinically correct in many cases and showed time-saving potential. While medical accuracy and usefulness varied across cases and should be focused on in further developments, a significant workload reduction is likely. Our anonymization process showed that regulatory challenges in the context of AI with patient data can effectively be dealt with. In summary, this study highlights the promise of transformer-based LLMs in reducing administrative tasks in health care. It outlines a pipeline for integrating LLMs into European Union clinical practice, emphasizing the need for careful implementation to ensure efficiency and patient safety.

## Introduction

In modern health care, administrative tasks have seen a sharp rise, escalating clinician workloads and increasing the risk of burnout and potential patient hazards due to an overload of work. [1,2] This trend has led to a critical demand within the medical community for effective and reliable assistance with documentation tasks. In parallel, the world of artificial intelligence (AI) has witnessed the development of large language models (LLMs) by organizations such as OpenAI and Anthropic and big tech companies like Google. These models have demonstrated their capability for generating coherent, human-like text that closely resembles professionally written content. However, out-of-the-box solutions like ChatGPT and similar models are, in many cases, not viable for immediate implementation in medical settings. This is due to several factors, including “refusal” due to built-in safety checks, the lack of alignment with local treatment guidelines, and the absence of detailed domain-specific knowledge [3,4].

To address these limitations, the concept of fine-tuning LLMs has emerged. Fine-tuning involves training existing language models on specialized data sets to enhance their performance in specific domains. Haghigi et al [5] demonstrated that training LLMs with specialized ophthalmic data improves their effectiveness in clinical decision-making and patient education. Their EYE-Llama models, pretrained on ophthalmology-specific data sets and fine-tuned on diverse question-answering data sets, outperformed baseline Llama 2 and ChatDoctor models, achieving results comparable to ChatGPT.

Van Veen et al [6] demonstrated that LLMs can outperform medical experts in summarizing clinical text across tasks like radiology reports, patient questions, and progress notes. Their study found that in most cases, summaries generated by adapted LLMs were equivalent or superior to those produced by human experts. This suggests that integrating LLMs into clinical workflows could significantly reduce the documentation burden on clinicians, thus allowing them to dedicate more time to patient care.

This study examines the feasibility of using adapted LLMs to automate medical report writing, specifically focusing on generating epicrises, or summaries. In our ophthalmology reports, there are three main sections: (1) patient history and diagnosis, (2) clinical findings, and (3) the epicrisis, which synthesizes the key information. We identified the epicrisis as the most suitable target for LLM-based assistance, as it synthesizes and consolidates information from the earlier sections. This reduces the risk of hallucinations by the model since the content is already documented and can be cross-referenced. In contrast, sections such as history and findings present more uncertainty and are more prone to variability or missing data, making them vulnerable to confabulation [7].

Epicrises are the most promising targets for automating the generation of medical reports. Heilmeyer et al [8] already provided valuable insights into fine-tuning LLM on premises for epicrisis generation to fulfill a similar task. To achieve this, different LLMs were fine-tuned on local servers to generate parts of medical reports, with varying degrees of success. We hypothesized that large commercial LLMs, such as OpenAI, hosted on capable infrastructure could outperform the local LLMs with limited resources and may be more accessible to sites with lower local resources. To our knowledge, OpenAI does not fully disclose the infrastructure on which the models are hosted, but it is known to use the Microsoft Azure Cloud and have access to a state-of-the-art graphics processing unit (GPU), such as NVIDIA H100, and server infrastructure [9].

Transferring medical reports to the United States for the purpose of improving medical report writing using an LLM poses significant challenges due to stringent legal and regulatory requirements. The General Data Protection Regulation (GDPR) in the European Union (EU) sets high standards for the protection of personal data, including medical information. According to the GDPR, the transfer of personal data to a third country, such as the United States, is only legal under certain conditions. Two primary approaches can be taken to make this transfer feasible: anonymizing the data beforehand or using Standard Contractual Clauses (SCCs) in combination with obtaining individual consent [8,10-12].

First, fully anonymizing the text under the legal basis of “legitimate interest” and validating the results as part of a Data Protection Impact Assessment (DPIA) is one approach. Once the data are anonymized, they no longer fall under the stringent regulations governing personal data, thus facilitating the transfer.

Alternatively, the use of SCCs or Binding Corporate Rules (BCRs), coupled with obtaining individual consent, can provide a lawful basis for the transfer. The invalidation of the Privacy Shield framework by the European Court of Justice (Schrems II decision) necessitates these legal mechanisms, along with additional safeguards, to ensure an adequate level of data protection [12]. Furthermore, ensuring compliance with the Health Insurance Portability and Accountability Act (HIPAA) is essential for any US entity handling health information. These regulations require comprehensive risk assessments, stringent data protection measures, and maintaining transparency with data subjects [13,14].

By either fully anonymizing the data before transfer or utilizing SCCs in combination with individual consent, the process of leveraging LLMs for enhancing medical report writing can be successfully navigated within the regulatory framework.

Other groups have already investigated approaches to anonymize clinical report data. Wiest et al [15] presented an LLM-based anonymization pipeline that can effectively deidentify medical free-text data, facilitating secure data sharing for medical research. This approach used a different locally installed LLM to anonymize clinical text data.

Therefore, the primary aim of this feasibility study was to evaluate the potential of a fine-tuned LLM, specifically OpenAI's Curie model, to automate the generation of ophthalmic epicrises. We hypothesized that such a model, when fine-tuned on a large, anonymized data set of clinical reports, could generate accurate and clinically useful summaries, thereby demonstrating the potential to reduce clinician documentation workload. This study specifically sought to assess the feasibility of this approach, the quality of the generated epicrises through both human and automated evaluations, and potential time savings, all while ensuring compliance with data protection regulations like GDPR.

## Methods

### Overview

Our approach involved obtaining quality training data from a large pool of consecutive medical letters available in our electronic database, which numbered close to 60,000. The data had no inclusion criteria to avoid introducing bias and to reflect real-world medical letters. Only high-quality medical letters that were reviewed by both a resident and an attending physician were included. Incomplete letters with missing fields were excluded.

### Anonymization Process

The following custom anonymization algorithm was developed and applied to all letters. In the first step, we extracted the data using an SQL query that selectively recalled the content of the letters but not any identifying data, such as names and addresses. The patient ID, however, was kept alongside the textual data for the second step. Here, we handled the rare cases where identifying information had been placed within the letters’ contents, such as a reference to the patient’s name and/or precise temporal information. This was done by explicitly replacing the patient's first name and surname with specific placeholders, using the patient ID to look up all identifying words to be eliminated from the document. The third step involved applying several regular expressions to capture date/time values, third-party names, and postal addresses. All occurrences were substituted with specific constant placeholders to preserve the semantic context and grammar.

This approach ensured that there was only a comparably small remaining text corpus containing only the information needed for the individual visit and not the whole patient history. This makes the anonymization process more consistent. Deanonymization in this case can only be assumed to be possible for highly individual cases and rare diseases. To ensure the robustness of our anonymization process during a DPIA, we conducted a rigorous evaluation. A randomly selected sample of 300 anonymized letters was independently reviewed by 2 expert investigators who were blinded to the original content. Neither reviewer was able to identify any residual personal data, demonstrating the algorithm's effectiveness in protecting patient privacy. Following this successful validation, our data protection officer approved the transfer of the anonymized data set to OpenAI.

We opted for this custom-made approach because it offers excellent reproducibility and explainability, giving us a high level of control over the anonymization process to ensure its quality. While other anonymization methods, including those using LLMs, exist, they do not provide the same degree of control, making our approach more reliable for our needs [15].

### Training and Fine-Tuning Process

OpenAI offers self-service fine-tuning for selected models from the GPT-3 family. We opted for the second powerful base model named “Curie” at the time of training since we wanted to assess the feasibility of our approach and not get a false-negative outcome due to selecting a base model with insufficient capabilities [16]. The training data set comprised a total of 90 million tokens, which were derived from approximately 60,000 medical letters.

We conducted a simple completion training in which the full medical letter, excluding the epicrisis, served as the prompt and the epicrisis as the corresponding completion. We did not use any instructions during training.

The fine-tuning of our model was achieved by using the OpenAI fine-tuning API via the official command line tools. The fine-tuning methodology of OpenAI is not publicly disclosed to the best of our knowledge. However, the low pricing and 24/7 availability of the fine-tuned model make it likely that some kind of adaptor technology is used instead of fully fine-tuning the base model. Hyperparameters, such as learning rate and batch size, are chosen automatically by the OpenAI command line tools.

The overall cost of fine-tuning totaled approximately 500 € (US $581). The fine-tuned model was hosted by OpenAI and accessed via a private key using the official API during the study period. At the time of publishing this article, the model has now been deactivated because its base model was depreciated by OpenAI, although the API remains operational [16].

### Integration Into a Testing Environment for a Global Assessment

We built a custom evaluation software tool. In this step, the physicians were prompted to evaluate the quality of sequential model-generated epicrises for anonymized data from real patients that had not been used to train the model. The testing physicians’ personal data were not collected, ensuring maximal data privacy without creating conflicts with employee rights. This study was performed during the period between calendar weeks 11 and 35 in 2023. The evaluation was based on a single Likert scale question ranging from “disturbing” to “excellent.”

### Structured, Multi-Item Human Evaluation in a Holdout Set of the Training Data

To evaluate the model’s performance and potential for real-world application, we conducted a comprehensive study involving a systematic clinician rating of epicrises generated from a set of random, anonymized historical report prompts. This seemed to be the best approach to evaluate the model’s performance and to estimate its potential benefits.

The human evaluation was performed using another custom intranet-based Python-programmed web application. Four experienced physicians, with professional experience ranging from 1 to 20 years, used the evaluation tool to rate the output of the generated epicrises in different metrics that are described later in the study. The physicians were given a guideline on how to evaluate each aspect.

### Estimating the Time-Saving Potential

We also developed a method to estimate the time savings for physicians when using AI-generated epicrises. In our multidimensional evaluation tool, we included a section where users copied and pasted the AI-generated epicrisis and revised it to match the quality of a gold-standard human-written version. We measured the time taken for this task. Prior to this, users completed a test to assess their writing speed. These results were then used to calculate the estimated time savings per epicrisis. This approach was intended to account for the possibility that there are mistakes of any kind in the AI-generated text, but just adapting the text would still be a benefit in terms of time consumption compared to writing a completely new epicrisis.

### Structured Multi-Item Automated Evaluation Using 2 LLMs

We implemented an automated evaluation technique where the outputs from LLMs were assessed based on several parameters. This evaluation was conducted using the commercially available OpenAI GPT-3.5 Turbo model, alongside Google’s open-source Gemma2 model (27B), to provide more nuanced and unbiased results [17,18]. We chose 2 different models to avoid potential bias, as the epicrises being evaluated were generated by an OpenAI model, and using only models from the same ecosystem could introduce bias.

The OpenAI model was accessed through the official API, with the total cost for evaluating all 86 letters amounting to approximately $0.20 USD. These costs were privately funded, and there were no conflicts of interest. The Gemma2 model was accessed via Ollama and installed locally on an Apple MacBook Pro (M3 Processor, 24GB RAM; Apple Inc).

For each evaluation, the medical report, human-written epicrisis (as the gold standard), and AI-generated epicrisis were provided as inputs. The models were prompted to assess whether the AI-generated text was medically accurate, formally correct, provided a time-saving advantage for clinicians, and was overall useful. Responses were requested on a 5-point scale ranging from “I disagree” to “I completely agree,” aligning closely with the human evaluation to ensure consistency.

Prompt engineering played a crucial role in obtaining meaningful results. The prompting was achieved using state-of-the-art techniques and libraries, such as LangChain. A total of 86 randomly selected reports were evaluated by both the OpenAI and Gemma2 models, using the same reports as in the human evaluation to enhance repeatability and consistency. Our evaluation prompt was carefully designed to align with the other evaluation metrics, ensuring coherence across all assessments.

### Ethical Considerations

The Ethics Committee of the University of Freiburg (24-1370-Anfrage) confirmed that formal ethical approval was not required for this project, as it exclusively involved the use of fully anonymized data. The committee clarified that research using anonymized text data does not constitute human participant research under the current ethical guidelines. All research was conducted in adherence to relevant ethical guidelines and regulations, ensuring compliance with institutional and international standards.

Individual informed consent was not required as this study used only fully anonymized data, with no possibility of identifying individual patients. This approach was validated through a DPIA and approved by our institutional data protection officer.

### Data Analysis

Statistical analyses were conducted using Python (SciPy, NumPy, and Pandas). A 1-sample Wilcoxon signed-rank test was used to compare subjective ratings (eg, formal correctness, medical accuracy, and usefulness) against a hypothesized neutral midpoint of 2.5. Paired *t* tests were employed to compare time and length measurements (eg, manual vs AI-corrected writing durations, and character counts). As each AI-corrected epicrisis was compared against the original human-written version for the same medical report, these data were inherently paired. Agreements between human and automated assessments were evaluated descriptively, based on the percentage of categorical rating alignment.

## Results

### Results Of the 1-Dimensional Clinical Rating

**Figure 1:**
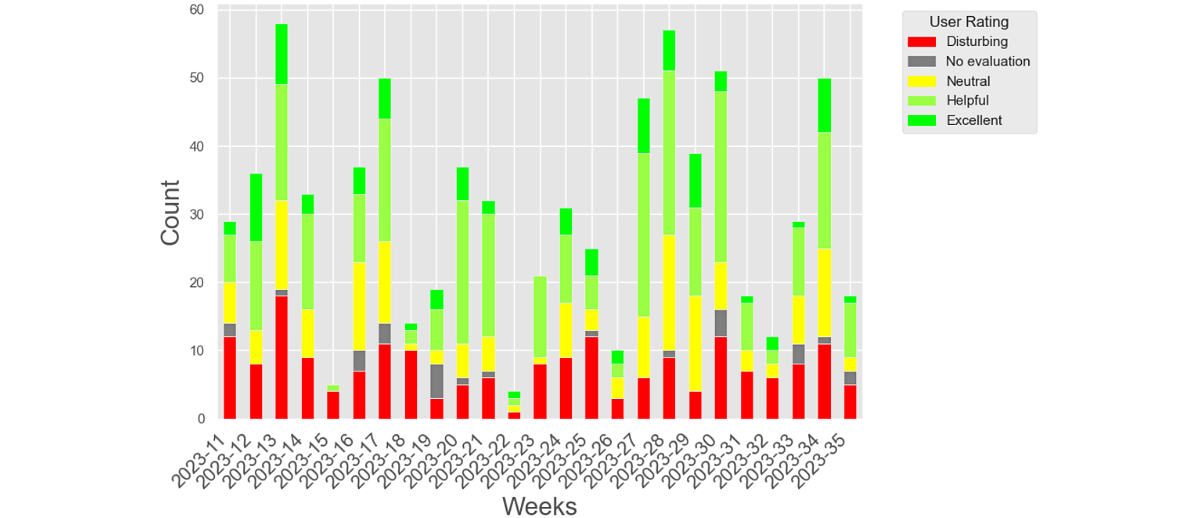
One-dimensional human evaluation recorded during the calendar weeks 11 to 35. The data were obtained over several weeks. Bad ratings are colored in red tones and good ratings in green tones .

In the real-world evaluation (n=769), the AI-generated epicrises received a mean rating of 1.08 (SD 1.38, coding for helpful) on a scale from –1 (disturbing) to 3 (excellent). Most ratings were positive, with 290 of 769 (37.7%) marked as helpful and 94 of 769 (12.2%) as excellent, while 194 of 769 (25.2%) were rated disturbing, indicating some polarization (Table 1). The results of the real-life clinical rating are shown in Figure 1. Although a 1-sample Wilcoxon signed-rank test did not show a statistically significant deviation from the neutral benchmark of 1 (*W*=83,038; *P*=.87), this may be attributable to the broad rating scale and inherent variability in real-world clinical presentations. Given the favorable distribution and positive trend in responses, the results still suggest that the model’s output was generally perceived as useful and well-accepted in everyday practice.

**Table 1:** Distribution of the 1-dimensional human evaluation. The largest percentage was evaluated to be useful.

Rating	Count (N=769), n (%)
Disturbing	194 (25.2)
No evaluation	30 (3.9)
Neutral	161 (20.9)
Helpful	290 (37.7)
Excellent	94 (12.2)

### Results of the Manual Evaluation

The physician rating was performed by 4 different physicians with clinical experience ranging from 1 year to more than 20 years. The ratings were performed as explained earlier in the Methods section. Table 2 shows the summarized numerical results of the evaluation.

**Table 2:** Overall physician rating of the AI^a^-generated epicrises. The ratings of all 4 physicians are summarized.

Evaluation metric	1: I disagree (n=107), n (%)	2: I partly agree (n=147), n (%)	3: I mostly agree (n=235), n (%)	4: I completely agree (n=254), n (%)
Formally correct	12 (11.2)	8 (5.2)	48 (20.4)	118 (46.5)
Medically correct	32 (29.9)	48 (32.7)	63 (26.8)	43 (16.9)
Useful	33 (30.8)	47 (32)	67 (28.5)	38 (15)
Advantage in time	30 (28)	44 (29.9)	57 (24.3)	55 (21.7)

^a^AI: artificial intelligence.

**Figure 2:**
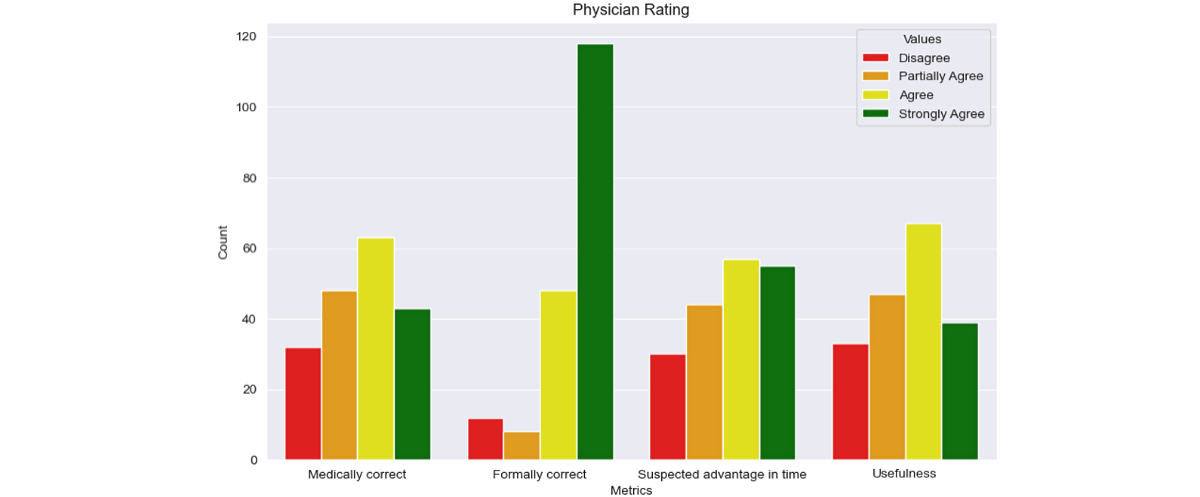
Physician rating of artificial intelligence (AI)-generated epicrises .

The proportion of evaluations representing a helpful AI written epicrisis was in all cases bigger than the proportion representing a rather disturbing or useless one. Figure 2 shows a different approach to visualizing the results grouped for the different metrics to experience a more nuanced view. The evaluations are very balanced for most metrics. In the case of the metric *formally correct*, there was a bigger tendency toward positive evaluations. Wilcoxon signed-rank tests were used to evaluate whether physician ratings differed significantly from a neutral midpoint of 2.5 on the 4-point rating scale. The neutral midpoint of the scale was 2.5, representing a neutral evaluation. The 1-sample Wilcoxon signed-rank test was used to assess if ratings significantly deviated from this midpoint. *Formally correct* was rated significantly higher than neutral (*W*=1686; *P*<.001), reflecting strong agreement on structural quality. *Advantage in time* was also rated significantly above the midpoint (*W*=6564; *P*<.01), indicating that clinicians perceived a clear time-saving benefit. In contrast, ratings for *medically correct* (*W*=7456*;* *P*=.08) and *usefulness* (*W*=7652.5; *P*=.18 did not significantly differ from neutral, suggesting more variable impressions regarding clinical accuracy and usefulness while still showing a quantitative tendency. All statistical results can be found in Table 3.

**Table 3:** Statistical results for the physician ratings.

Metric	Median	*W* statistic	*P* value	Significant *(P<*.05)
Medically correct	3	7456	.08	No
Formally correct	3	1686	<.001	Yes
Useful	3	7652.5	.18	No
Advantage in time	3	6564	.003	Yes

### Calculation of Possible Time Advantage

As previously described, we added a way of estimating a possible benefit in time consumption for our study. The time the tester needed to correct the computer-generated epicrisis was measured. The time to write the human-written epicrisis was calculated by dividing the length of the human-written epicrisis by the user’s individual writing speed. The average time to rewrite the human epicrisis was 109.52 seconds, while the average time to correct the AI-generated epicrisis was 54.25 seconds, suggesting a time advantage of 56 seconds per epicrisis. The exact results are shown in Table 4.

**Table 4:** Times and lengths of the epicrises evaluated by 4 different physicians (N=69).

Statistic	Real epicrisis time (seconds)	Corrected epicrisis time (seconds)	AI^a^-generated length (characters)	Real length (characters)	Corrected length (characters)
Mean	109.52	54.25	330.43	501.07	352.22
SD	53.3	63.34	115.42	243.5	135.78
Min	11.35	0.77	125	52	118
25th percentile	73.14	21.94	261	335	255
Median	98.47	44.46	315	451	342
75th percentile	131.86	65.12	400	604	445
Max	261.4	499.86	689	1192	691

^a^AI: artificial intelligence.

What must be considered in this context is that there were significant differences in epicrisis length. To assess whether differences in documentation time were influenced by the length of the epicrises, we compared character counts across versions. AI-generated epicrises were significantly shorter than human-written ones (t_68_=–6.10; *P*<.001), and the corrected AI versions also remained significantly more concise (t_68_=–6.65; *P*<.001). These results suggest that the AI system may support more efficient and focused documentation by generating concise summaries of relevant information. This may be particularly beneficial for nonnative speakers, as it helps reduce linguistic complexity while preserving clinical content. The differences are portrayed in a boxplot that can be found in Multimedia Appendix 1. Additionally, this may be due to a person’s information advantage when writing, since not all information can be inferred from the letter above. A paired *t* test comparing the time required for generating human-written epicrises versus correcting AI-generated ones revealed a statistically significant reduction in time with AI assistance (t_68_=3.39*; P*=.001). On average, correcting the AI-generated epicrises was notably faster, suggesting a potential efficiency gain in documentation workflows. This result supports the hypothesis that AI-assisted documentation can meaningfully reduce the time burden for clinicians. All exact statistical results are shown in Table 5.

**Table 5:** Statistical results for time and length comparisons.

Comparison	Mean (SD)	Test type	*t* test (df)	*P* value
Time: human-written vs corrected AI^a^	109.52 (53.3) vs 54.25 (63.3) s	Paired *t* test	3.39 (68)	<.001
Length: human-written vs AI-generated	501 (243.5) vs 330.43 (115.42) characters	Paired *t* test	–6.10 (68)	<.001
Length: human-written vs corrected AI version	501 (243.5) vs 352.22 (135.78) characters	Paired *t* test	–6.65 (68)	<.001

^a^AI: artificial intelligence.

### Results of the Automated Evaluation

The automated evaluation showed a tendency for medium values but demonstrated an overall good correspondence with the human evaluation from earlier. Notably, the automated AI evaluation was more hesitant to assign extreme values. The exact results of the evaluations can be seen in Table 6, and they show good overall correspondence. Automated LLM assessments generally aligned with human ratings, with over 52% (356/679) of LLM responses and 66% (489/743) of human ratings falling into the top 2 agreement categories. While this supports overall consistency, direct statistical comparisons (eg, interrater reliability) are not appropriate, as LLMs are not human raters. The evaluation serves as proof of concept for future refinement.

**Table 6:** AI^a^-generated evaluation of the epicrises. The evaluation was conducted using 2 LLMs^b^. The first was OpenAI’s GPT-3.5 Turbo model, accessed through the OpenAI API. The second was a locally run Gemma2 (Google LLC), accessed through Ollama.

Metric		Disagree	Partially agree	Agree	Strongly agree
**OpenAI ChatGPT 3.5 Turbo**					
	Medically correct	0	62	22	1
	Formally correct	2	18	64	1
	Suspected advantage in time	4	28	44	8
	Usefulness	0	63	22	0
**Google Gemma2**					
	Medically correct	0	48	37	0
	Formally correct	1	9	74	1
	Suspected advantage in time	3	18	53	11
	Usefulness	1	66	18	0

^a^AI: artificial intelligence.

^b^LLM: large language model.

## Discussion

### Principal Findings

Our study had 2 primary objectives. First, we aimed to develop a proof of principle for medical report writing assistance under optimal conditions, providing valuable insights for future developments that could be implemented within EU regulatory frameworks. Second, we sought to assess the potential degree of workload reduction for clinicians.

To achieve the goal of exploring the full potential of current LLM technology in medical report writing, we deliberately chose to use OpenAI's high-resource data centers and powerful models. Although this approach may have limitations for routine usage in the EU due to GDPR constraints, our work may also be directly useful for sites outside the EU without the infrastructure to fine-tune and host LLMs on premises.

By using OpenAI resources, we were able to demonstrate a best-case scenario for LLM-assisted medical report writing. Our results show that under these conditions, there was a significant potential for reducing the administrative burden on clinicians. The real-life clinical ratings, where more than 50% of evaluations found the AI-generated epicrises helpful or excellent, suggest that such a system could substantially decrease the time and effort required for documentation tasks.

The physician ratings and automated evaluations further support this potential for workload reduction. The high scores in categories such as *advantage in time* and *useful* indicate that AI-generated epicrises could save valuable clinician time, allowing for more focus on direct patient care.

The estimation for writing time revealed a potential savings of approximately 56 seconds (nearly 50%) per medical report. Given that physicians typically handle about 20 reports daily, this could lead to a significant reduction in workload. Our study successfully demonstrated that our approach is a viable solution to achieve these goals, showcasing the potential of LLMs in medical documentation. Additionally, we developed an effective anonymization algorithm that complies with the stringent data protection standards of European law for data transfer to non-EU countries. This accomplishment is significant, as it facilitates collaboration on sensitive patient data while maintaining compliance with GDPR. Our anonymization process, replacing sensitive patient data with placeholders, was crucial in providing the LLM with a high-quality text corpus to work with.

### Comparison With Prior Work

Our findings align with and build upon several key studies in this field. There are now several studies that show that the use of LLMs can be helpful in reducing physician workload [6,19].

Van Veen et al [6] demonstrated that adapted LLMs could outperform medical experts in summarizing clinical texts, with their generated summaries often rated as equivalent or superior to human-written ones. This study adopted a broader approach across various medical fields. Heilmeyer et al [8] explored the use of open-source LLMs hosted on premises for a similar task, focusing on the viability of local, privacy-preserving solutions. Other approaches that could also be combined with our work in a helpful context include that of Kernberg et al [20], who used LLMs to create medical notes from audio files. Singh et al [21] successfully used LLMs to create discharge summaries and operative notes. We believe this demonstration suggests that our approach could potentially be applied in a broader context while working with the same techniques.

### Limitations

We recognize that the time-saving potential is merely an estimate, and actual results may vary in practice, which we could not evaluate at this stage. A standardized assessment of the time-saving potential is methodically challenging to quantify.

In addition, it is crucial to note that these results represent an ideal scenario, while real-world implementation may present additional challenges. This might include retraining the fine-tuned models or developing models that are capable of online training to adapt to changing guidelines and state-of-the-art medical treatment. The development of models with reinforcement learning may be a good approach and should be considered in further research.

While our anonymization process was conducted to fulfill the high data privacy standards according to the laws in Germany and Europe, it must still be considered that complete anonymization is difficult to achieve. There are cases of very rare diseases, such as corneal or retinal dystrophies, or other individual conditions that could potentially be identified if supplementary data were available.

Our training corpus has limitations in terms of size and domain diversity, which may affect the model’s adaptability. We observed that the model performs best on very common, standardized cases in our daily clinical routine, such as pterygium, keratoconus, and age-related macular degeneration. On the other hand, the model sometimes struggles with more complex cases involving individualized treatment plans. This has motivated us to continue in-house model development to improve the training corpus and retrain a new model in the future. It is also worth noting that the model may perform differently when applied to other medical disciplines. Ophthalmology, as a specialized field, may present challenges for generally trained models, while areas like internal medicine, which benefit from more extensive training data, may see better results.

While our approach is relatively straightforward to implement, significant challenges remain. Many hospitals do not have structured access to medical reports and still use unstructured formats like Microsoft Word, complicating automated data processing. Although our model setup is simple, delivering data to APIs such as OpenAI in a structured JSON format is crucial. For clinics that can achieve this, the training process becomes easier compared to developing in-house hardware and software for model fine-tuning. However, considerable domain knowledge is still required for effective implementation, which may be lacking in many hospitals.

Evaluating the performance of language models in medical contexts is inherently challenging, which is why we implemented a variety of metrics to assess our model’s performance. We aimed to create a set of possibilities that can be used to evaluate the performance of LLMs. This mainly relied on human ratings in different contexts. We also included an AI auto-evaluation as a proof of concept that could be applied in other contexts with bigger sample sizes that are difficult to evaluate manually. Since the human and the AI auto evaluation showed good alignment, we believe this approach can be used for similar tasks in the future.

Several factors must be considered when evaluating our project and identifying areas for future research. The rapid advancement of generative AI requires ongoing reevaluation.

One limitation in this context is the fact that this study was conducted using the OpenAI Curie model, which has since been deprecated. While the specific model is no longer state-of-the-art, the primary contribution of our work lies in the demonstration of a feasible end-to-end pipeline. The methodologies and principles established here are directly transferable to newer, more powerful models, and we are actively conducting ongoing research with these models.

Additionally, other groups within our hospital have achieved success using on-premises solutions for this task. However, the training and fine-tuning processes remain challenging because of the demand for highly qualified personnel as well as hardware requirements. Our study did not perform a direct head-to-head comparison, and the hypothesis that commercial models outperform local ones remains a topic for future empirical validation.

### Conclusion

In conclusion, our study demonstrates the significant potential of LLMs in medical report generation under optimal conditions, provides a quantitative assessment of possible workload reduction for clinicians, and highlights the challenges of implementing such systems within the EU's regulatory framework. The proof of principle established here sets a benchmark for what is achievable with the current LLM technology. Future research should focus on developing equally powerful solutions that can be deployed on premises or within EU-based cloud services, ensuring both high performance and full compliance with the GDPR. We hope that this study will guide the development of EU-compliant solutions capable of matching or exceeding this performance level while significantly reducing the administrative burden on health care professionals—allowing physicians to focus rather on patient care than on medical documentation. Our work represents one step toward achieving this goal [3].

## Appendix

Multimedia Appendix 1 Calculated time to write the human-written epicrises with the respective writing speed of the tester and lengths of the epicrises in characters.

## Abbreviations

AI: artificial intelligence
BCR: Binding Corporate Rule
DPIA: Data Protection Impact Assessment
EU: European Union
GDPR: General Data Protection Regulation
GPU: graphics processing unit
HIPAA: Health Insurance Portability and Accountability Act
LLM: large language model
SCC: Standard Contractual Clause

## Data Availability

The data sets used and/or analyzed during this study are available from the corresponding author upon reasonable request.

## References

[1] Robertson SL, Robinson MD, Reid A (2017). Electronic health record effects on work-life balance and burnout within the I3 population collaborative. J Grad Med Educ.

[2] Overhage JM, McCallie D (2020). Physician time spent using the electronic health record during outpatient encounters: a descriptive study. Ann Intern Med.

[3] Yu P, Xu H, Hu X, Deng C (2023). Leveraging generative AI and large language models: a comprehensive roadmap for healthcare integration. Healthcare (Basel).

[4] Karabacak M, Margetis K (2023). Embracing large language models for medical applications: opportunities and challenges. Cureus.

[5] Haghighi T, Gholami S, Sokol JT, Kishnani E, Ahsaniyan A, Rahmanian H, Hedayati F, Leng T, Alam MN (2025). EYE-Llama, an in-domain large language model for ophthalmology. iScience.

[6] Van Veen D, Van Uden C, Blankemeier L, Delbrouck J, Aali A, Bluethgen C, Pareek A, Polacin M, Reis EP, Seehofnerová A, Rohatgi N, Hosamani P, Collins W, Ahuja N, Langlotz CP, Hom J, Gatidis S, Pauly J, Chaudhari AS (2024). Adapted large language models can outperform medical experts in clinical text summarization. Nat Med.

[7] Huang L, Yu W, Ma W, Zhong W, Feng Z, Wang H, Chen Q, Peng W, Feng X, Qin B, Liu T (2025). A survey on hallucination in large language models: principles, taxonomy, challenges, and open questions. ACM Trans Inf Syst.

[8] Heilmeyer F, Böhringer D, Reinhard T, Arens S, Lyssenko L, Haverkamp C (2024). Viability of open large language models for clinical documentation in German health care: real-world model evaluation study. JMIR Med Inform.

[9] NVIDIA Hopper GPUs expand reach as demand for AI grows. NVIDIA News.

[10] Bradford L, Aboy M, Liddell K (2020). International transfers of health data between the EU and USA: a sector-specific approach for the USA to ensure an 'adequate' level of protection. J Law Biosci.

[11] EU Regulatory Data Protection: International data transfer rules for non-personal data. DLA Piper.

[12] Hallinan D, Bernier A, Cambon-Thomsen A, Crawley FP, Dimitrova D, Medeiros CB, Nilsonne G, Parker S, Pickering B, Rennes S (2021). International transfers of personal data for health research following Schrems II: a problem in need of a solution. Eur J Hum Genet.

[13] International transfers -. European Data Protection Supervisor.

[14] Liss J, Peloquin D, Barnes M, Bierer BE (2021). Demystifying for the cross-border transfer of clinical research data. J Law Biosci.

[15] Wiest I, Leßmann ME, Wolf F, Ferber D, Treeck Mv, Zhu J, Ebert MP, Westphalen CB, Wermke M, Kather JN (2025). Deidentifying medical documents with local, privacy-preserving large language models: the LLM-anonymizer. N Engl J Med AI.

[16] OpenAI Platform.

[17] Ollama Gemma2.

[18] Gemma 2 is now available to researchers and developers. Google Blog.

[19] Chen C, Liao C-T, Tung Y-C, Liu C-F (2024). Enhancing healthcare efficiency: integrating ChatGPT in nursing documentation. Stud Health Technol Inform.

[20] Kernberg A, Gold JA, Mohan V (2024). Using ChatGPT-4 to create structured medical notes from audio recordings of physician-patient encounters: comparative study. J Med Internet Res.

[21] Singh S, Djalilian A, Ali MJ (2023). ChatGPT and ophthalmology: exploring its potential with discharge summaries and operative notes. Semin Ophthalmol.

