# Supplementary figures and images for: Streamlining Ophthalmic Documentation With Anonymized, Fine-Tuned Language Models: Feasibility Study
Source: Interact J Med Res. 2025 Nov 26;14:e72894. doi: 10.2196/72894 (PMC12696452; doi:10.2196/72894)

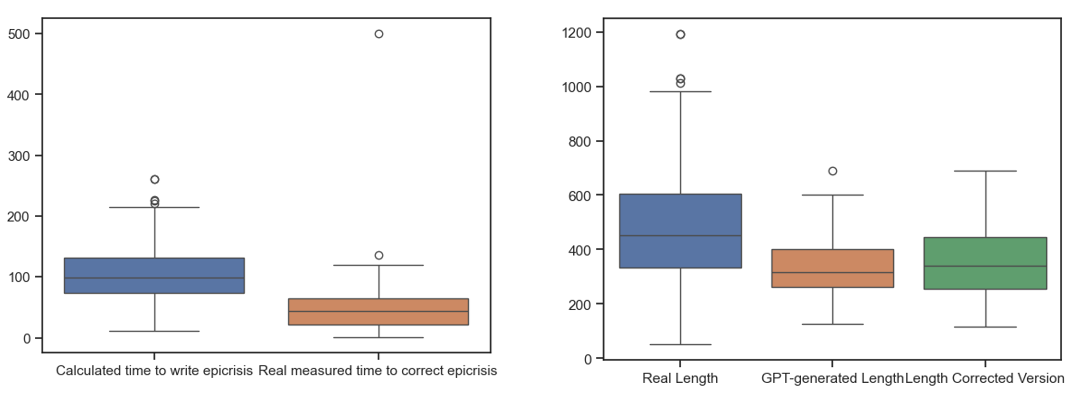

Supplement: Multimedia Appendix 1 [file ijmr_v14i1e72894_app1.png]
